# Supplementary material for: The interactome of intact mitochondria by cross-linking mass spectrometry provides evidence for coexisting respiratory supercomplexes
Source: Mol Cell Proteomics. 2017 Dec 8;17(2):216–32. doi: 10.1074/mcp.RA117.000470 (PMC5795388; doi:10.1074/mcp.RA117.000470)
Supplement: Supplemental Data [file supp_RA117.000470_133922_0_supp_23978_fzffwf.zip › spectra_annotation/mito_spectra_annotation/86-1-8-1-14-1.pdf]

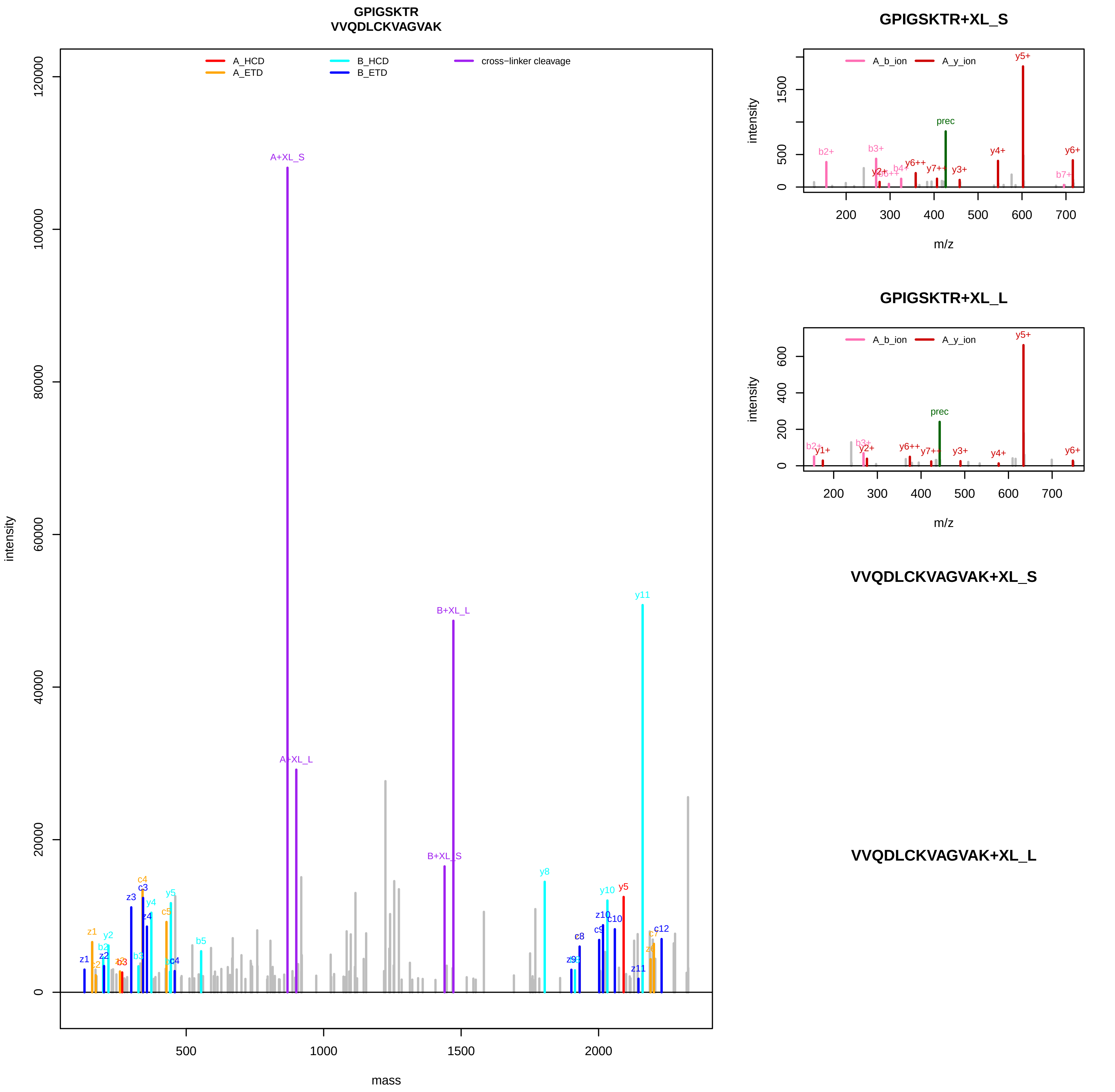
GPIGSKTR
VVQDLCKVAGVAK
A_HCD
A_ETD
B_HCD
B_ETD
cross−linker cleavage
A+XL_S
A+XL_L
B+XL_L
B+XL_S
y11
y8
y10
y5
z10
c10
c9
c8
z11
c12
z1
y2
b2
z2
z3
c4
c3
y4
z4
y5
c5
b5
c4
intensity
mass
GPIGSKTR+XL_S
A_b_ion
A_y_ion
prec
y5+
y4+
y6+
b2+
b3+
b4+
y6++
y7++
y3+
y2+
b7+
m/z
GPIGSKTR+XL_L
y1+
VVQDLCKVAGVAK+XL_S
VVQDLCKVAGVAK+XL_L
